# Supplementary material for: A Modified AUC for Training Convolutional Neural Networks: Taking Confidence Into Account
Source: Front Artif Intell. 2021 Nov 30;4:582928. doi: 10.3389/frai.2021.582928 (PMC8670229; doi:10.3389/frai.2021.582928)
Supplement: Supplementary file 1 [file Presentation1.pdf]

Appendix A: Model Architectures and Training Settings

We utilized the PyTorch version 1.9 deep learning framework to design and train the models. Tables 12-14 show details of model architectures and optimization settings for each experiment. More information abour the settings such as kernel size, stride, padding, and dilation is available in PyTorch documents[1].

Table 12. Architecture and settings of the MNIST experiments

Architecture	
(cnn1): Conv2d(1, 16, kernel_size=(5, 5), stride=(1, 1))<br>(relu1): ReLU()<br>(maxpool1): MaxPool2d(kernel_size=2, stride=2, padding=0, dilation=1)<br>(cnn2): Conv2d(16, 32, kernel_size=(5, 5), stride=(1, 1))<br>(relu2): ReLU()<br>(maxpool2): MaxPool2d(kernel_size=2, stride=2, padding=0, dilation=1)<br>(fc1): Linear(in_features=512, out_features=2, bias=True)	
Settings	
Batch Size	1
Optimizer	Stochastic Gradient Descent (SGD)[20]
Loss function	Weighted BCE
Learning rate	0.0002
Momentum	0.8
L2 Penalty	0.001
Maximum number of epochs	50

Table 13. Architecture and settings of the PCa experiments

Architecture	
(cnn1): Conv2d(6, 16, kernel_size=(7, 7), stride=(1, 1))<br>(maxpool1): MaxPool2d(kernel_size=2, stride=2, padding=0, dilation=1)<br>(relu1): ReLU()<br>(dropout1): Dropout(p=0.1, inplace=False)<br>(cnn2): Conv2d(16, 32, kernel_size=(5, 5), stride=(1, 1))<br>(maxpool2): MaxPool2d(kernel_size=2, stride=2, padding=0, dilation=1)<br>(relu2): ReLU()<br>(dropout2): Dropout(p=0.1, inplace=False)<br>(cnn3): Conv2d(32, 64, kernel_size=(4, 4), stride=(1, 1))<br>(maxpool3): MaxPool2d(kernel_size=2, stride=2, padding=0, dilation=1)<br>(relu3): ReLU()<br>(dropout3): Dropout(p=0.1, inplace=False)<br>(fc1): Linear(in_features=1024, out_features=256, bias=True)<br>(fc2): Linear(in_features=256, out_features=64, bias=True)<br>(fc3): Linear(in_features=64, out_features=2, bias=True)	
Settings	
Batch Size	1
Optimizer	SGD
Loss function	Weighted BCE
Learning rate	0.0001
Momentum	0.8
L2 Penalty	0.001
Maximum number of epochs	50

[1] https://pytorch.org/docs/stable/index.html

Table 14. Architecture and settings of the BraTS experiments

Architecture	
(cnn1): Conv2d(3, 16, kernel_size=(7, 7), stride=(1, 1))<br>(maxpool1): MaxPool2d(kernel_size=2, stride=2, padding=0, dilation=1)<br>(relu1): ReLU()<br>(dropout1): Dropout(p=0.1, inplace=False)<br>(cnn2): Conv2d(16, 32, kernel_size=(5, 5), stride=(1, 1))<br>(maxpool2): MaxPool2d(kernel_size=2, stride=2, padding=0, dilation=1)<br>(relu2): ReLU()<br>(dropout2): Dropout(p=0.1, inplace=False)<br>(cnn3): Conv2d(32, 64, kernel_size=(4, 4), stride=(1, 1))<br>(maxpool3): MaxPool2d(kernel_size=2, stride=2, padding=0, dilation=1)<br>(relu3): ReLU()<br>(dropout3): Dropout(p=0.1, inplace=False)<br>(fc1): Linear(in_features=36864, out_features=256, bias=True)<br>(fc2): Linear(in_features=256, out_features=64, bias=True)<br>(fc3): Linear(in_features=64, out_features=2, bias=True)	
Settings	
Batch Size	1
Optimizer	SGD
Loss function	Weighted BCE
Learning rate	0.0001
Momentum	0.85
L2 Penalty	0.001
Maximum number of epochs	50

Appendix B: AUCPlus

In python, sklearn[2] is the most renowned package containing functions for AUC and ROC. We have developed a function called AUCPlus published in GenuineAI[3] package. For the case described in Table 15, Fig 11 shows the ROC curves created by sklearn as well as AUCPlus. Sklearn uses derivatives to change values of threshold. This results in unpredictable number of points in the ROC curve. In other words, sklearn retains minimum number of points needed for drawing an ROC curve. Although in theory at each fixed TPR level, minimum FPR is desired, there are cased we may prefer to have a margin. For example, if there is a noise which imposes randomness and uncertainty, we will move away from the minimum FPR. In those cases, AUCPlus will be helpful.

[2] https://scikit-learn.org/

[3] https://pypi.org/project/GenuineAI/

The main limitation of sklearn is that it only returns thresholds and ROC. Our function, on the other hand, returns a dataframe containing thresholds along with TP, TN, FP, FN, TPR, FPR, Specificity, Accuracy, Precision, and F1 score at each t.

Table 15. Randomly generated probabilities and Real Values (N=10)

Actual values	0	0	0	0	1	1	1	1	0	1
sorted Probabilities	0.1	0.2	0.3	0.4	0.5	0.6	0.7	0.8	0.9	1

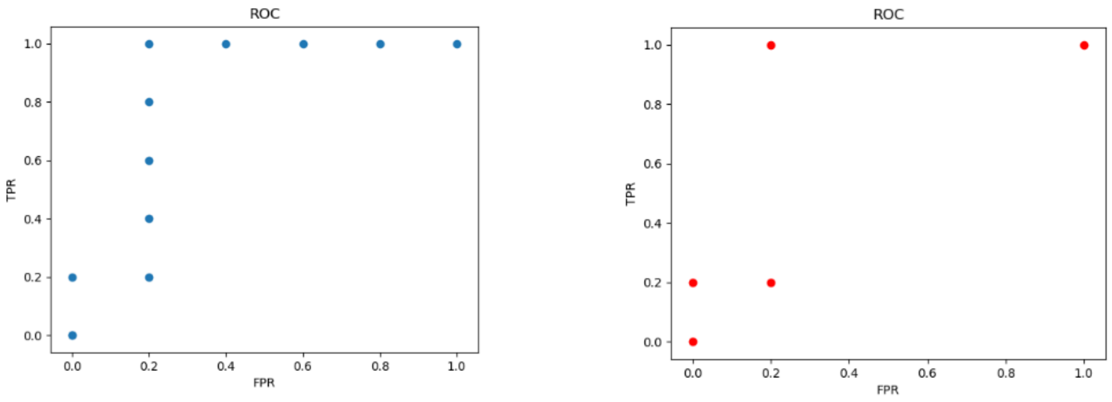

(a)GenuineAI. AUCPlus

(b) sklearn.metrics.roc_curve

Fig. 11. SKLearn ROC vs GenuineAI ROC (N=10)
